# Integrated MicroRNA–mRNA Sequencing Analysis Identifies Regulators and Networks Involved in Feline Hypertrophic Cardiomyopathy

**DOI:** 10.3390/ijms26146764

**Published:** 2025-07-15

**Authors:** Jessica Joshua, Jeff L. Caswell, Anja Kipar, M. Lynne O’Sullivan, Geoffrey Wood, Sonja Fonfara

**Affiliations:** 1 Department of Clinical Studies, Ontario Veterinary College, University of Guelph, 50 Stone Road East, Guelph, ON N1G 2W1, Canada; jjoshua@uoguelph.ca; 2 Department of Pathobiology, Ontario Veterinary College, University of Guelph, 50 Stone Road East, Guelph, ON N1G 2W1, Canada; jcaswell@uoguelph.ca (J.L.C.); gewood@uoguelph.ca (G.W.); 3 Vetsuisse Faculty, Institute of Veterinary Pathology, University of Zurich, Winterthurerstrasse 268, CH-8057 Zurich, Switzerland; anja.kipar@uzh.ch; 4 Department of Companion Animals, University of Prince Edward Island, 550 University Ave, Charlottetown, PE C1A 4P3, Canada; mosullivan@upei.ca

**Keywords:** cat, heart, remodeling, HCM, atrium, ventricle, pathway, inflammation, fibrosis, angiogenesis

## Abstract

Cardiac remodeling in feline hypertrophic cardiomyopathy (HCM) is poorly understood. To investigate underlying molecular mechanisms, we determined microRNA–mRNA interactions, regulatory networks, and upstream regulators using left ventricle (LV) and left atrium (LA) mRNA and microRNA sequencing datasets from cats with HCM and controls. Upstream regulators, molecules, and pathways associated with ischemia, inflammation, fibrosis, and cellular changes were observed in the HCM heart. In both the HCM LV and LA, TNFα, IL1β, and TGFβ were identified as upstream regulators, along with FGF23, THBS4, and FAMB177 as the top increased molecules. Relevant microRNAs included upstream regulator miR-132, enriched miR-124-3p, miR-122b-3p, miR-146-5p (HCM LV and LA), miR-370, miR-1185-5p, miR-12194-3p (HCM LV), miR-153-3p, miR-185-5p, and miR-185-3p (HCM LA). Macrophage pathways were activated, and granulocyte and agranulocyte adhesion and diapedesis were the most connected pathways. The HIF1α signaling pathway in the HCM LV, upstream regulators miR-1-3p and miR-204, and reduced miR-29 and miR-122-5p suggest cardioprotective mechanisms. Observed in the healthy heart and suspected to be involved in cardiac homeostasis were upstream regulators miR-96, inhibited WNT3A and miR-145, as well as miR-140-5p, miR-141-3p, miR-208b-3p, and miR-885-3p. This study provides further insights into the pathogenesis of HCM, and identifies the factors involved in the maintenance of a healthy LV and LA.

## 1. Introduction

Hypertrophic cardiomyopathy (HCM) is the most prevalent heart disease in cats [1,2]. Characterized by primary hypertrophy of the left ventricle (LV) and diastolic dysfunction, feline HCM is considered a model for human HCM [3,4]. Though a genetic basis for HCM has been suspected [5,6,7], disease penetrance and progression are variable and only little is known about the pathogenesis in cats. To improve the lack of knowledge, we recently carried out microRNA and mRNA sequencing of the LV and left atria (LA) from healthy cats and cats with HCM and identified disease-specific and chamber-associated mRNA and microRNA profiles [8,9].

MicroRNAs are well-known to play key roles in health and their dysregulation is associated with cardiovascular disease [10,11]. In the human healthy heart, microRNAs including miR-1, miR-16, miR-27b, miR-30d, miR-126, miR-133, miR-143, miR-208 and the let-7 family of microRNAs have been identified at all stages of development and are important for virtually all physiological processes involved in cardiac maintenance [12,13,14]. MicroRNAs are also known to be regulators of cardiac remodeling in mouse models and humans [15]. Studies investigating myocardial microRNA expression in patients with HCM found 66 microRNAs to be associated with the disease [16]. Similarly, our study on cats with HCM revealed 80 differently expressed microRNAs in the LV and 37 in the LA of the HCM heart compared to the healthy heart counterparts [9]. These included microRNAs aligned with HCM in humans, such as miR-21, miR-96, miR-122-5p, miR-132, miR-139, miR-146b, miR-208b, and miR-708 [17]. In our mRNA sequencing study, we observed 412 differently expressed mRNAs in the HCM LV and 1207 mRNAs in the HCM LA, which comprised genes that had been reported in hearts from humans with HCM such as THBS4 (thrombospondin 4), FMOD (fibromodulin) and IL18 (interleukin 18), and genes that were functionally unreported in the heart, such as KLHL33 (kelch like family member 33), FAM177B (family with sequence similarity 177 member B), and THRSP (thyroid hormone responsive) [8].

The altered expression of microRNAs leads to changes in target mRNA expression, indicating that integrating microRNA and mRNA profiles may provide a comprehensive and unique insight into the molecular mechanisms of a disease. In human HCM, network-based studies on microRNA–mRNA interactions have shown involvement in extracellular structure organization, organ growth, phagosome and melanoma pathways as well as actin filament and stress fiber formation, and calcium ion binding [18,19,20,21]. We hypothesized that determining microRNA–mRNA interactions of the HCM feline heart and hearts from cats without cardiac disease would provide key information about mediators and regulatory networks involved in the HCM pathogenesis. The objective of the study was to perform an integrated analysis of mRNA and microRNA sequencing datasets of the LV and LA from cats with HCM and healthy cats using Ingenuity Pathway Analysis (IPA).

## 2. Results

### 2.1. Differentially Expressed Myocardial mRNAs and microRNAs in Feline HCM and Health

Comparing the cats’ LV and LA mRNA and microRNA sequencing datasets GSE275971 [8,9], we identified the same 215 differentially expressed genes (DEG) and 15 differentially expressed microRNAs (DEM) in the HCM LV and LA when compared with their healthy counterparts (Table 1). For the regional comparison, the same 716 DEG and 17 DEM were found in the LV compared to the respective LA of both the healthy and HCM heart (Table 1).

### 2.2. MicroRNA–mRNA Interactions and Associated Pathways in the Heart from Cats with HCM

To investigate potential microRNA–mRNA interactions associated with HCM, we looked at microRNA–mRNA pairs, the resulting pathways (PWs), and associated regulators in the HCM LV and LA compared with the controls as well as the HCM LV compared with the HCM LA. We found 191 microRNA–mRNA interactions in the HCM LV and 244 in the HCM LA. In total, 223 microRNA–mRNA interactions were detected when the LV and the LA datasets were compared in HCM (Supplementary Tables S1a,b and S2a,b).

**Figure 1:**
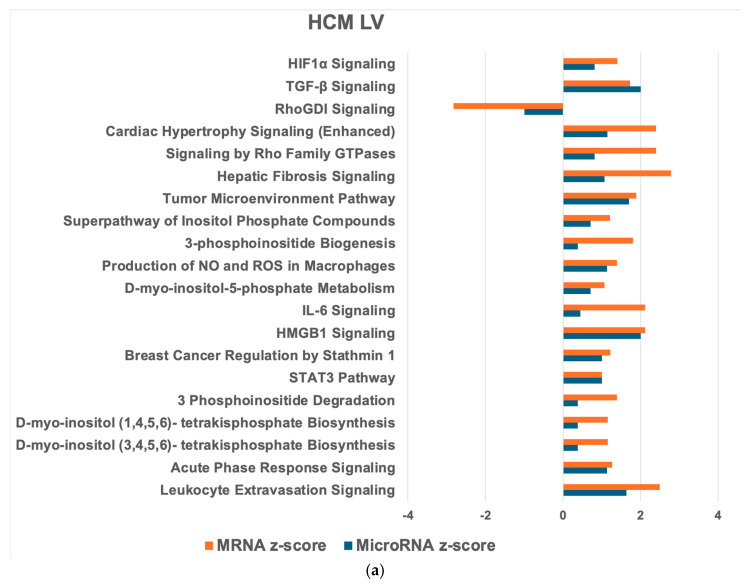

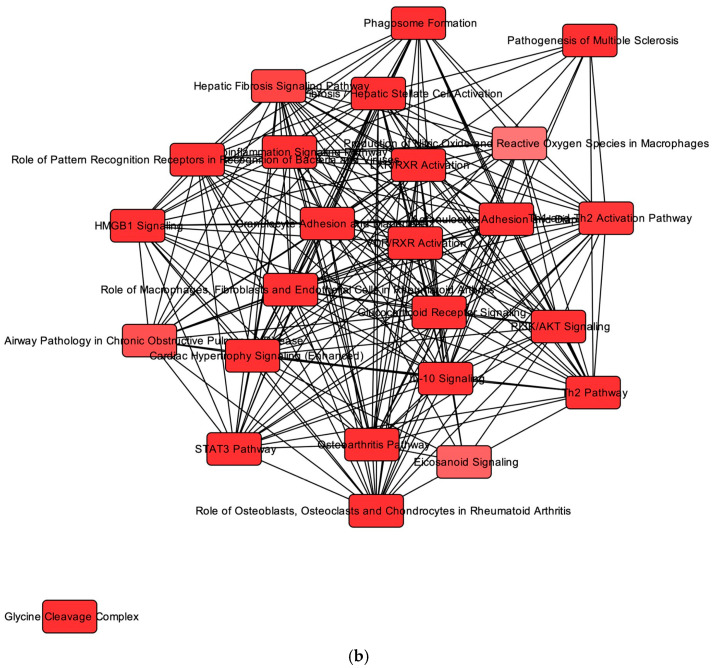
IPA pathway (PW) and network analysis of genes in the HCM left ventricle (LV) compared to the healthy LV (hLV) that overlapped from the mRNA and microRNA target datasets. Top 20 PWs with similar expression (**a**) and associated microRNA target networks (**b**) in the mRNA and microRNA target datasets for HCM LV compared to hLV. For the PWs, the x-axis shows the z-score (mRNA = orange, microRNA = blue) and the y-axis shows the name of the IPA PW. For the network, each node (red box) represents a PW and the lines connecting the PW represents the microRNA target genes that are significant in the dataset. HCM: Hypertrophic cardiomyopathy; IPA: Ingenuity PW Analysis.

In both the HCM LV and LA, the most downregulated microRNA was miR-122-5p, which targets EGLN3 (egl-9 family hypoxia inducible factor 3), a factor associated with HIF1α signaling, which we identified as the most activated PW in the HCM LV (Figure 1a). Enriched in the HCM LV and LA were miR-122-3p, miR-124-3p, and miR-132. These microRNAs target genes associated with inflammation, fibrosis, hypertrophy and cAMP signaling, and metabolic PWs. Also enriched was miR-146-5p, which targets IL1F10 (also known as IL38), a top reduced molecule in the HCM LV and LA (Supplementary Tables S1a,b and S2a,b).

Found only in the HCM LV were reduced miR-31-3p and -5p, miR-135a-5p, miR-218-5p, and miR-204-5p. These microRNAs were associated with genes involved in fibrosis and inflammatory PWs. Decreased miR-218-5p was associated with reduced B3GAT1 (also known as CD57) and miR-204-5p with enriched IL1R1 (also known as ST2) and EPHA5, which are top molecules in the HCM LV (Supplementary Table S1a,b).

The microRNA with the most increase in the HCM LV was miR-344d-3p targeting CLDN10 (claudin 10), linked with agranulocyte adhesion and diapedesis, granulocyte adhesion and diapedesis, and the leukocyte extravasation signaling PWs (Supplementary Table S1a). Additionally enriched microRNAs were miR-370-5p and -3p, miR-1185-5p targeting FGF13, and miR-12194-3p targeting HORMAD2 and LRRC4B, top reduced molecules in the HCM LV (Supplementary Tables S1a and S3a,b).

HCM LA-specific enriched microRNAs were miR-185-3p and miR-153-3p, which target genes involved in apelin, mTOR, hypertrophy, inflammation, and the metabolic signaling PWs (Supplementary Table S2a).

The regional comparison confirmed the relevance of miR-1185-5p and miR-12194-3p in the HCM LV as well as higher concentrations of miR-122b-3p and miR-124-3p in the HCM LA (Supplementary Tables S3a,b and S4a,b). It further identified a higher concentration of miR-1-3p in the HCM LV than LA (Supplementary Table S3a). MiR-1-3p was associated with TAC1, ANPEP (among the HCM LV top reduced molecules), reduced IGF1, and increased KCNJ2 (Supplementary Table S3a,b).

### 2.3. Overlapping mRNA and microRNA Pathways and Networks in the Heart of Cats with HCM

**Figure 2:**
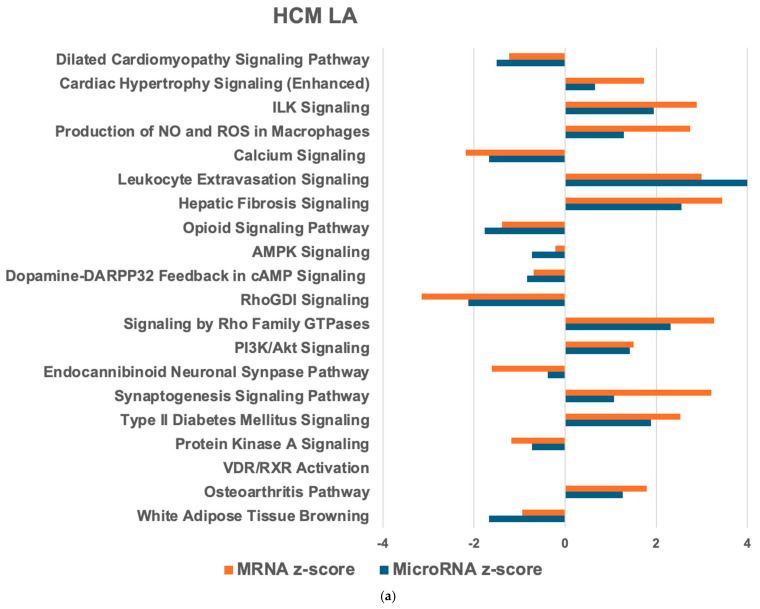

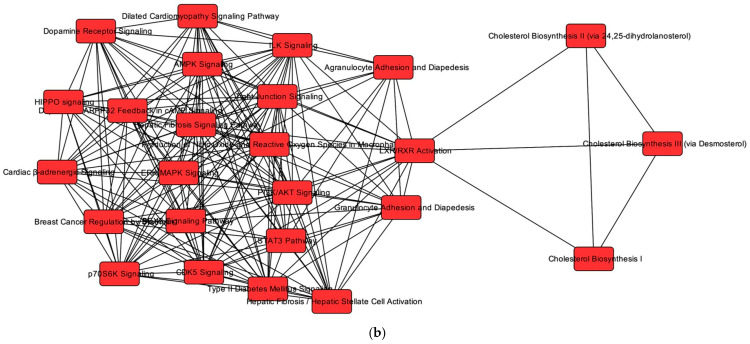
IPA PW and network analysis of genes in the HCM left atrium (LA) compared with the healthy LA (hLA) that overlapped from the mRNA and microRNA target datasets. Top 20 PWs with similar expression (**a**) and associated microRNA target networks (**b**) are shown for the HCM LA compared to hLA. For explanation of the graphs, see legend of Figure 1.

**Figure 3:**
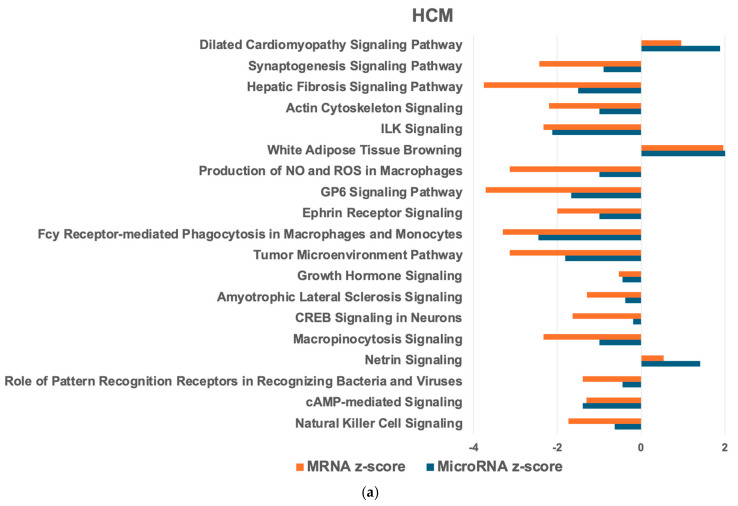

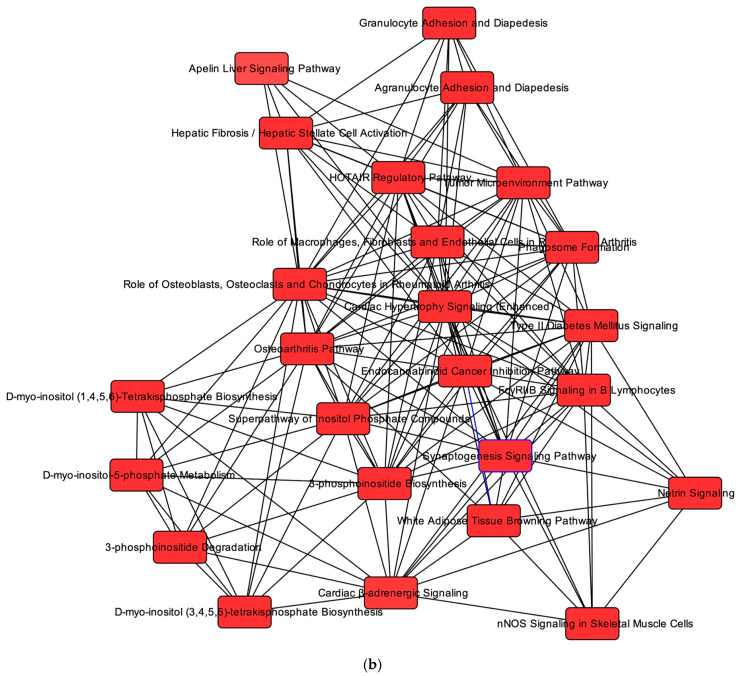
IPA PW and network analysis of genes in the HCM heart that overlapped from the mRNA and microRNA target datasets. Top 20 PWs with similar expression (**a**) and associated microRNA target networks (**b**) in the HCM LV compared to the HCM LA. For explanation of the graphs, see legend of Figure 1.

Next, we compared mRNA and microRNA target PWs in the HCM heart with the healthy heart to identify the HCM heart-relevant PW (Figure 1a and Figure 2a). In the HCM LV, 269 mRNA PWs and 91 microRNA target PWs were detected, of which 45 were common in both. For the HCM LA, 231 mRNA PWs and 146 microRNA target PWs were observed, with 118 common PWs. Within the HCM heart, 260 mRNA and 101 microRNA target PWs were identified, with 94 shared PWs. Most of these PWs were reduced in the HCM LV compared with the HCM LA (Figure 3a). The top activated PWs in the HCM LV compared with the healthy LV (hLV) were HIF1α signaling and TGFβ signaling (Figure 1a), while in the HCM LA, cardiac hypertrophy signaling, ILK signaling, and the production of nitric oxide (NO) and reactive oxygen species (ROS) in macrophages were most significantly upregulated (Figure 2a).

For the PW network analysis in the HCM LV and LA, we applied IPA. In both the HCM LV and LA (compared to the healthy counterparts), most connections were found between granulocyte adhesion and diapedesis as well as agranulocyte adhesion and diapedesis (13 targets; Figure 1b and Figure 2b). Cardiac hypertrophy signaling, hepatic fibrosis signaling PWs (10 targets), and inflammatory signaling PW (Figure 1b) were also highly connected in the HCM LV. In the HCM LA, major associations comprised hepatic fibrosis/hepatic stellate cell activation and hepatic fibrosis signaling (10 targets), cAMP signaling, and ERK/MAPK signaling (11 targets; Figure 2b). Comparing the HCM LV and LA, the largest cluster of connections was within the inositol PW (15 targets; Figure 3b).

### 2.4. Regulators of Networks and Target Molecules in the Heart from Cats with HCM

We further identified upstream (upRegs) and master regulators (mRegs) of networks and target molecules in the HCM heart. The top upRegs in both the HCM LV and LA, were beta-estradiol, dexamethasone, TNFα, IL1B, IL6, IFNG, AGT (angiotensinogen), TGFβ, IGF1, P38 MAPK, and forskolin, supporting the relevance of inflammation and fibrosis in the HCM heart. In the HCM LV, activated CSF1 and STAT3, and in the HCM LA, FGF2, NOTCH1, and inhibited PPARG and ADIPOQ were observed as top upRegs.

The top mRegs in the HCM LV included activated TH1 cytokine with 17 target-connected regulators and inhibited α2M-TNFα, etanercept, a TNFα inhibitor, miR-29, and miR-132. A total of 21 molecules were involved in cellular assembly and organization, 18 were associated with cardiac dysfunction, cardiac fibrosis, as well as cellular movement and inflammatory response.

In the HCM LA, the top causal network mRegs included PSMC5 (proteasome 26S subunit, ATPase 5), forskolin, and factors involved in CREB signaling. Forskolin had the highest number of participating target-connected regulators, with 67 in total. Downstream effect networks included cell proliferation, survival and differentiation, and cardiovascular disease with 29 molecules, cellular movement with 28 molecules, and carbohydrate metabolism, cell death and survival, and post-translational modification had 27 molecules. The top molecules in the HCM LV and LA included FGF23, THBS4, NEFM, IL1RL1, FAM177B, miR-122-5p, IL1F10, THRSP, NR4A3, and B3GAT1 (Supplementary Tables S1b and S2b).

For the regional comparison (HCM LV vs. HCM LA), miR-1-3p was identified as an activated upReg. The top mRegs of the HCM heart were D-2-amino-5-phosphonovaleric acid, cannabinol (a key regulator of inflammation), the ACE-inhibitors omaprilat and gemoprilat, and miR-204-5p. Downstream effect networks included molecular transport, carbohydrate metabolism, lipid metabolism, and small molecule biochemistry. The top molecules in the HCM LV are listed in Supplementary Table S3b.

### 2.5. MicroRNA–mRNA Interactions and Associated Pathways in the Healthy Feline Heart

We further characterized constitutive microRNA–mRNA pairs and associated PW in the healthy heart, comparing the hLV with the hLA. Overall, 190 microRNA–mRNA pairs were identified in the LV compared to the LA. The top enriched microRNAs in the hLV included miR-190a-3p, miR-106a-5p, miR-124-3p, miR-140-5p, and miR-18a-5p, while miR-141-3p, miR-181c-3p, miR-181a-5p, and miR-135a-1-3p were the top decreased (Supplementary Table S4a). Enriched miR-190a-3p targets SMCO1, which is essential for muscle structure and function and was also targeted by miR-18a-5p, CCL8, and FGF18, associated with granulocyte and agranulocyte adhesion and diapedesis (CCL8), as well as actin cytoskeleton and cardiac hypertrophy signaling (FGF18) (Supplementary Table S4a,b). Reduced miR-141-3p targets genes associated with PWs involved in inflammation (TAC1), BMP and TGFβ signaling (PITX2), and metabolism (PMP2) (Supplementary Table S4a).

### 2.6. Overlapping mRNA and microRNA Pathways and Networks in the Healthy Feline Heart

**Figure 4:**
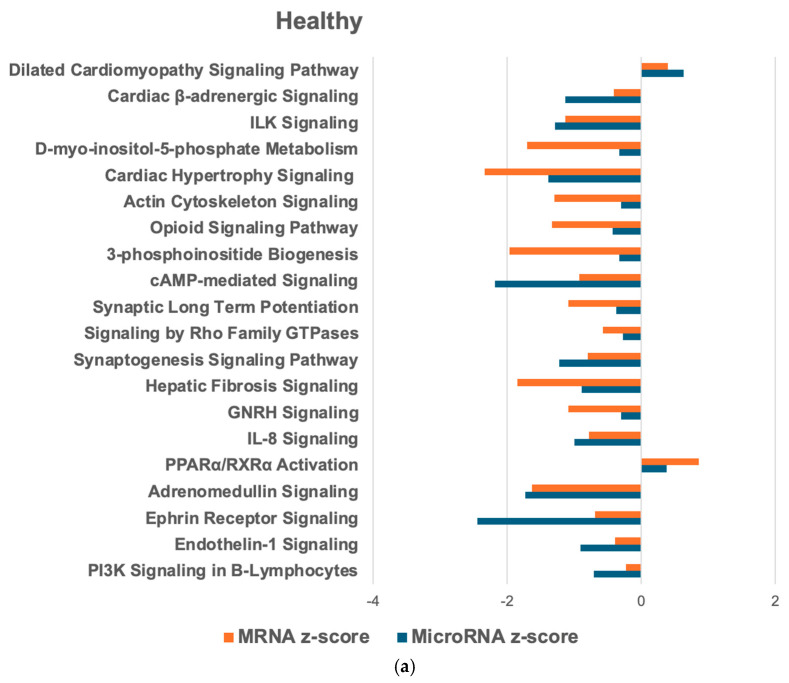

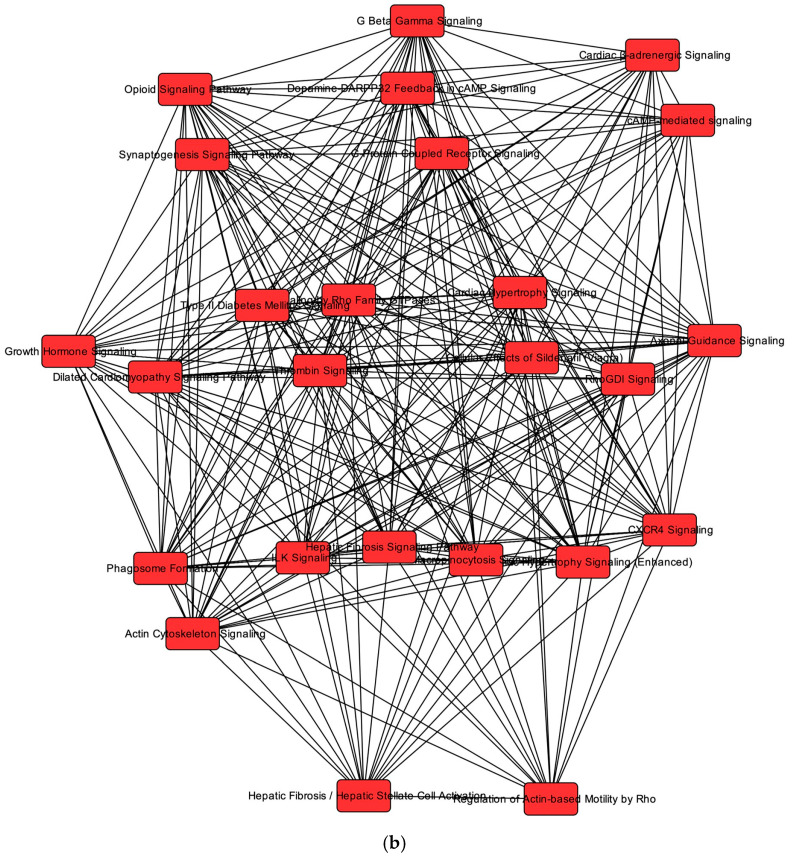
IPA PW and network analysis of genes in the healthy heart that overlapped from the mRNA and microRNA target datasets. Top 20 PWs with similar expressions (**a**) and associated microRNA target networks (**b**) in the hLV compared to the hLA. For explanation of the graphs, see legend of Figure 1.

In the hLV compared to the hLA, we identified 250 mRNA PWs and 228 microRNA target PWs, with 140 common PWs. Similarly to the HCM heart, most PWs were less abundant in the LV compared to the LA (Figure 4a). PW network analysis revealed that in the hLV, most connections were present between RhoGDI signaling and signaling by Rho Family GTPases (17 targets), G-protein coupled receptor signaling and cAMP-mediated signaling (16 targets), and thrombin signaling and CXCR4 signaling (15 targets; Figure 4b).

### 2.7. Regulators of Networks and Target Molecules in the Healthy Feline Heart

Similarly to the HCM heart, the upRegs included beta-estradiol, dexamethasone, TNFα, IL1B, IL6, IFNG, AGT, TGFβ, IGF1, forskolin, WNTA3, and CREB1. However, contrary to the HCM heart, most of these regulators were inhibited in the hLV. Causal network analysis identified inhibited WNT3A as the top mReg, followed by molecules associated with the CREB signaling. MiR-96-5p and TRSP1 were activated mRegs, while KRAS, IL11RA, and miR-145-5p were inhibited mRegs. The WNT3A network comprised 24 target-connected regulators. Overall, 32 molecules were involved in cell death and survival functions, 30 molecules in lipid metabolism, molecular transport, and nucleic acid metabolism, and 28 molecules were associated with cardiovascular disease, cardiovascular system development and function, and organ morphology. Molecules enriched in the hLV included IRX4, miR-208b-3p, MYL3, MYH7, MLANA, and SMCO1, and those enriched in the hLA were BARX2, MYL4, PITX2, CLEC3A, NPR3, SUSD3, and miR-885-3p (Supplementary Table S4b).

## 3. Discussion

In this study, we performed an integrated analysis using mRNA and microRNA datasets from the same samples to determine regulatory mechanisms involved in the PWs found to be altered in the feline HCM heart. By analyzing overlapping expression patterns, we identified regulators, molecules, microRNAs, and PWs generally associated with feline HCM as well as the expression of cardiac chamber-specific genes and microRNAs. The results indicate a complex remodeling process, involving progressive, compensatory, and cardioprotective processes.

We detected 191 microRNA–mRNA interactions in the HCM LV and 244 in the HCM LA when compared with their healthy counterparts. These were associated with activated and highly connected inflammatory, hypertrophy, and metabolic PWs as well as acute phase response and ERK/MAPK signaling. For human HCM, 731 mRNA–microRNA interactions have been reported, and the same PWs were observed, although these were downregulated [22]. We suspect these discrepancies are associated with the disease stage; the datasets used for our study were from cats with progressed HCM, exhibiting gene expression patterns similar to those obtained from human hearts with advanced HCM and DCM [23]. Another study on human HCM using an integrated microRNA–mRNA analysis found 20 overlapping DEG and DEM associated with enriched PWs comparable to our results, supporting the relevance of these PWs in the HCM pathogenesis [19].

We identified TNFα, IL1β, TGFβ, AGT, and P38 MAPK as upRegs in the HCM LV and LA, and ACE-inhibitors and miR-132 as mRegs. This indicates their key role in cardiac remodeling and confirms the importance of inflammation and fibrosis in the disease process. The relevance of TNFα in the HCM LV was supported by the inhibited mRegs alpha2M-TNFα complex and TNFα inhibitor etanercept. MiR-132 is known to be involved in cardiac fibrosis and hypertrophy [24,25]. The top increased molecules FGF23, THBS4, and FAMB177 and the activated TGFβ and fibrosis PWs further support this interpretation. FGF23 stimulates pro-fibrotic factors, induces pro-hypertrophic genes, contributes to endothelial dysfunction, and is associated with inflammatory macrophages [26,27]. THBS4 was recently reported as an activated fibroblast marker in human HCM and DCM hearts [23], and FAM177B has been reported as an upregulated marker in M1 macrophages [28]. Interestingly, IL1F10 (also known as IL38), an anti-inflammatory cytokine, was found to be decreased in both the HCM LV and LA. In mouse models of myocardial ischemia, reduced IL38 has been shown to promote an inflammatory macrophage response and stimulate pro-inflammatory cytokines [29,30]. IL38 was associated with miR-146-5p, which has previously been identified as relevant microRNA in the pathogenesis of human HCM [20]. Similarly, NR4A3, a transcription factor with anti-inflammatory function, was reduced. NR4A3 is associated with a reduction in macrophages in animal models and humans with myocardial infarction and cardiac hypertrophy [31]. The absence of macrophage PWs in the healthy heart and an activation in the HCM heart supports the relevance of macrophages in feline HCM. Furthermore, granulocyte and agranulocyte adhesion and diapedesis were the most connected PWs in the HCM LV and LA.

Regional differences in inflammation markers were observed. In the HCM LV, CSF1, which promotes macrophage maturation and M2 macrophage polarization [32,33], was identified as an activated upReg. ARG2, which stimulates macrophage inflammatory responses, was also activated, alongside increased miR-1185. MiR-1185 targets FGF13, a marker of the tissue resident reparative CCR2-negative macrophages. This macrophage population was observed in the LV of cats with HCM and was one of four macrophage populations found in the LV from people with progressed HCM and DCM [23,34,35]. Furthermore, the HCM LA exhibited a higher activity of macrophage PWs than the HCM LV, and CD79A and CD3, a B-lymphocyte and pan T-cell marker, respectively, were among the top increased molecules in the HCM LA. These regional differences may be driven by ANPEP, which was higher in the HCM LA and associated with miR-1-3p. ANPEP is expressed on activated monocytes and endothelial cells and facilitates the transendothelial migration of monocytes in cell lines and mouse models [36]. B3GAT1 (also known as CD57), a marker for mature T-lymphocytes and associated with reduced miR-218-5p [37], was found to be reduced in the HCM heart. These results support the relevance of macrophages and involvement of diverse inflammatory cell populations in the remodeling processes in the LV and LA of cats with HCM.

Relevant microRNAs that we suspected to be involved in the HCM remodeling process were miR-124-3p and miR-122b-3p, both enriched in the HCM LV and LA. These microRNA are associated with inflammation, fibrosis, and metabolic changes. In the HCM LV, additional top-enriched miR-344d-3p, miR-323-3p, miR-1185-5p, miR-3118, and miR-12194-3p may contribute to these changes, as they influence gene expression related to inflammation, cytoskeleton, hypertrophy, fibrosis, angiogenesis, and metabolic signaling. MiR-12194-3p was associated with decreased HORMAD2, a signal responsive adapter mediating protein–protein interactions [38], and reduced LRCC4B, which results in cell proliferation in cancer cell lines [39]. Reduced miR-135a-5p in the HCM LV was associated with the upregulated molecule NEFM, a gene involved in WNT–catenin signaling, which may be related to the observed phenotypic changes in cardiomyocytes, inflammation, and oxidative stress [34].

In the LA HCM, miR-153-3p, which was present in the healthy LA and increased in the HCM LA, may contribute to the remodeling processes by inhibiting genes of apelin signaling pathways (known to have cardioprotective effects), and mTOR signaling, particularly mTORC1, whose activation leads to cardiac hypertrophy [40,41]. Similarly, enriched miR-185-5p was associated with increased IL7R and PWs involved in inflammation, cardiac hypertrophy, and B-cell development. The latter might be consistent with the upregulated molecule CD79A in the HCM LA. Among the inhibited top upRegs in the HCM LA were PPARG and ADIPOQ. ADIPOQ was also a top reduced molecule in the HCM LA and targeted by the enriched miR-185-3p. Reduction in these factors indicate inflammation, cell proliferation, fibrosis, endothelial, and metabolic dysfunction (reduced fatty acid oxidation and glucose uptake) [42,43].

Interestingly, our findings also revealed the presence of compensatory processes and several cardioprotective factors that may counteract the adverse remodeling processes in feline HCM. Forskolin, identified as mReg and upReg in the HCM LV and LA, suggests compensatory processes. It had the highest number of connected target regulators, and its downstream effect networks included cell proliferation, survival and differentiation, and cardiovascular disease. Forskolin is an activator of myocardial adenylate cyclase, which causes an increase in cAMP, a main second messenger that modulates cardiac function, inflammation, and metabolic processes [44,45]. The chronic activation of cAMP signaling is a known response to myocardial ischemia to compensate for cardiomyocyte degeneration and impaired cardiac function [46]. We suspect ischemia as an initiating or contributing event in HCM remodeling processes in cats [34,47]. This is supported by HIF1α signaling, which is activated in hypoxia, being the top activated PW in the HCM LV. Furthermore, the top reduced miR-31-5p and miR-122-5p were associated with activated EGLN3, a cellular oxygen sensor involved in HIF1α signaling [48]. The activated HIF1α signaling PW initiates the transcription of pro-survival genes promoting cell survival. UpRegs and mRegs with known cardioprotective effects in the HCM LV included miR-1-3p, miR-29, and miR-204, as well as miR-122-5p, which was the top reduced microRNA in the HCM LV and LA and is a known key microRNA in human HCM [20]. Inhibited miR-29, miR-204, and miR-122-5, along with miR-1-3p, have been reported to prevent cardiac hypertrophy and fibrosis and have anti-inflammatory properties in mouse models [49,50,51,52,53,54,55,56,57,58]. Specifically, miR-29 functions by suppressing WNT signaling [49], while miR-1-3p targets TAC1 [52,53,54]; both were reduced in the feline HCM LV. miR-204-5p was associated with increased molecules of IL1RL1 (also known as ST2) and EPHA5. ST2 is the ligand for IL33 and signals through MyD88. Both were activated upRegs in the HCM LV and were found to reduce cardiac fibrosis and hypertrophy in knock-out mouse models [55]. ST2 is further involved in the activation of inflammatory cells and expression of M2 markers on macrophages. Activated EPHA5 modulates cell adhesion and influences the cellular cytoskeleton, potentially contributing to the observed phenotypic cardiomyocyte changes in the cat HCM LV [34].

In the HCM LA, the top upRegs FGF2 and NOTCH1 might facilitate the transformation of progenitor cells into endothelial cells [59,60]. The presence of progenitor cell populations and angiogenesis in the HCM LV has been reported previously [34]. NOTCH1 is reported to promote proliferation and differentiation of cardiac progenitor cells [60] and to reduce oxidative stress as well as prevent cardiac fibrosis in myocardial ischemia [60].

The results for the healthy feline heart identified microRNAs, mediators, and PWs that regulate myocardial homeostasis. Interestingly, the upRegs that were involved in the feline HCM heart were also found in the healthy heart but were inhibited instead of activated. mRegs in the healthy heart were activated miR-96, a cardiac enriched microRNA, inhibited WNT3A and miR-145, and regulators of CREB signaling. These are involved in cell development, differentiation, and proliferation, as are the networks with the most targets: RhoGDI, Rho Family GTPases, cAMP-mediated signaling, CXCR4 signaling, cardiac hypertrophy, and fibrosis. RhoGDI, which is higher in the healthy heart than the feline HCM heart, inhibits the family of Rho GTPases which drive inflammation, fibrosis, and vascular remodeling in cardiac hypertrophy [61].

Further involved in cardiac maintenance were miR-18a-5p, miR-124-3p, miR-140-5p, and miR-141-3p. miR-18a-p5 in the hLV was associated with increased SMCO1, a gene that has recently been identified as cardiac enriched in humans [62]. miR-18a-5p has further been reported to have cardioprotective effects and is associated with cardiac aging [63,64]. The presence of miR-124-3p in the healthy heart, which was increased in the HCM heart, suggests a role in cardiac maintenance at lower concentrations, whereas its dysregulation in HCM may contribute to fibrosis and metabolic imbalance. mir-140-5p, which was more abundant in the hLV and HCM LV than the corresponding LA was associated with NPR3, a natriuretic peptide receptor relevant for cardiovascular homeostasis [65], and SUSD3 (involved in regulation of the cytoskeleton) in the hLA. mir-141-3p, the top reduced in the hLV and HCM LV, belongs to the miR-200 family, which is present in cardiomyocytes and endothelial cells and is involved in cell growth and survival, fibrosis, and angiogenesis [66]. This is consistent with miR-141-3p target genes: reduced PITX2 (associated with BMP and TGFβ signaling), TAC1 (a molecule that is associated adverse remodeling [52,67]), and ELAVL2 (an RNA binding protein that promotes translation and stabilizes MMP9 [68,69]), and increased MLANA (a T-cell antigen expressed in the heart [70,71]) and PMP2 (a fatty acid binding protein that is involved in lipid metabolism). miR-208b-3p, a LV-enriched microRNA that is involved in cardiac repair and regeneration, and miR-885-3p were confirmed as the top molecules in the hLV and hLA, respectively [9].

Limitations of this study include the potential influence of age on the results. The healthy cats from which the datasets used in this study were derived were significantly younger (1.5 years) than the cats with HCM (3–15 years) [8,9]. While this allowed the identification of the constitutive mRNA and microRNA profiles in the healthy cat heart, some results observed for cats with HCM might be associated with age and not HCM. Only male cats were included in this study, which limits the generalizability of the results to female cats. Additionally, the cats with HCM had progressed cardiac disease, which may restrict the applicability of the findings to earlier stages of HCM.

## 4. Materials and Methods

### 4.1. Data of Gene Expression and microRNA Profiles

**Figure 5:**
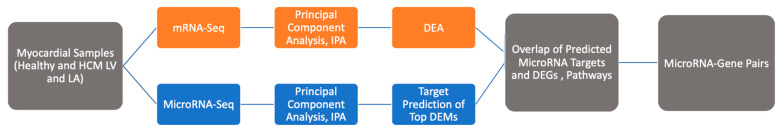
Flowchart of RNA-seq enriched PW analysis and microRNA–mRNA pairs prediction strategy. DEA: Differential Expression Analysis; DEGs: Differentially Expressed Genes; DEMs: Differentially Expressed MicroRNAs; HCM: Hypertrophic cardiomyopathy; LV: Left ventricle; LA: Left atrium; IPA: Ingenuity Pathway Analysis.

Datasets obtained from our mRNA and microRNA sequencing studies (GSE275971) were used for the current project [8,9]. The datasets originated from LV and LA tissue samples of 12 cats with HCM and 8 healthy cats, comprising 5 LV and 5 LA HCM as well as 5 LV and 5 LA healthy mRNA datasets along with 7 LV and 5 LA HCM as well as 8 LV and 8 LA healthy microRNA datasets. Paired LV and LA tissues from two cats with HCM were included in both datasets. The studies were reviewed and approved by the Animal Care Committee of the University of Guelph [8,9]. Analyses performed are summarized in Figure 5.

### 4.2. Differentially Expressed mRNAs and microRNAs

The differential expression of mRNA and microRNA between the HCM and the control groups (HCM LV vs. hLV; HCM LA vs. hLA), as well as between the LV and the LA (HCM LV vs. HCM LA; hLV vs. hLA), were conducted using R (edge R and DeSeq2 bioinformatic tools). The raw data were normalized using the trimmed mean of M-values method and standardized using the log2 transformation. Log2 fold change >1 and adjusted *p*-value < 0.05 were cut-offs for filtering DEG and DEM. Only microRNA targets that were also present in the mRNA dataset were included in the analysis. The top microRNA–mRNA pairs were identified for each comparison using microRNA target pairing and by listing those with mRNAs that were found to be the most significant in our dataset.

### 4.3. Pathway and Network Analysis

To analyze the PWs, molecules, and microRNA–mRNA interactions in the HCM and control hearts, we filtered the targets predicted by IPA from the microRNA dataset. The most abundant microRNAs from our differential expression analysis are expected to affect their target mRNAs; therefore, microRNAs with a negative correlation to the mRNA targets were chosen. In addition to microRNA–mRNA target pairing, the top canonical PWs and networks were identified. Upstream analysis was performed to identify upRegs (e.g., transcription factors, cytokines, kinases) that may be activated or inhibited and are directly linked to the genes in our datasets. Causal network analysis identified mRegs that were not directly linked to targets but had direct and indirect associations through intermediate regulators. The top upregulated and downregulated molecules were obtained from the analyzed dataset using IPA core analysis.

## 5. Conclusions

Our results revealed complex myocardial remodeling in feline HCM, including progressive, compensatory, and cardioprotective processes. Integrated mRNA–microRNA analysis identified upRegs and mRegs, molecules, and PWs in the HCM heart that supported the presence of ischemia, the role of inflammation and fibrosis, and indicated the involvement of macrophages and progenitor cells in the HCM heart, exhibiting regional differences.

TNFα, IL1β, TGFβ, AGT, P38 MAPK, and miR-132, were identified as upRegs and mRegs, and FGF23, THBS4, and FAMB177B as top molecules in both the HCM LV and LA. These factors are known to influence hypertrophy, fibrosis, endothelial function, and macrophage populations. Markers of inflammatory cell populations, in particular macrophages, were found, exhibiting regional differences. In the HCM LA, FGF2 and NOTCH1 might drive the transition of progenitor cells. MicroRNAs likely associated with the remodeling processes in the feline HCM heart, were increased miR-124-3p, miR-122b-3p, miR-132, and miR-146-5p in the HCM LV and LA; miR-344d-3p, miR-370, miR-1185-5p, and miR-12194-3p in the HCM LV; and miR-153-3p, miR-185-5p, and miR-185-3p in the HCM LA.

The presence of counter-regulatory mechanisms was observed. Forskolin was identified as a mReg and upReg and HIF1α signaling as the top activated PW in the HCM LV; both were associated with the attenuation of inflammatory, fibrosis, structural, and metabolic processes. These protective responses may be mediated by the upRegs and mRegs miR-29, miR-1 and miR-204, and reduced miR-122-5p.

In the healthy heart, several microRNAs and molecules likely contribute to cardiac homeostasis. These include the upRegs miR-96, inhibited WNT3A and miR-145, and miR-140-5p, miR-141-3p, miR-208b-3p, and miR-885-3p.

## Figures and Tables

**Table 1:** Overview of the transcriptome and miRNome datasets listing the number of significantly up- and downregulated differentially expressed genes and microRNAs in the HCM and healthy heart, including the overlap between up- and downregulated genes. All DEGs and DEMs that were log2FC +/− 1, FDR < 0.01 and FPKM > 3 were considered significant. Comparisons are shown for HCM-associated differences (HCM LV vs. Healthy LV, and HCM LA vs. Healthy LA) and regional differences (HCM LV vs. HCM LA, and Healthy LV vs. Healthy LA). Overlap refers to common genes or microRNAs found within both comparison groups.

Groups	Differentially Expressed Genes	Differentially Expressed microRNAs
HCM LV vs. Healthy LV	412 (290 UP, 122 DOWN)	80 (66 UP, 14 DOWN)
HCM LA vs. Healthy LA	1707 (724 UP, 983 DOWN)	37 (22 UP, 15 DOWN)
HCM LV vs. HCM LA	1513 (545 UP, 968 DOWN)	77 (52 UP, 25 DOWN)
Healthy LV vs. Healthy LA	1423 (507 UP, 916 DOWN)	37 (9 UP, 28 DOWN)
HCM Overlap (HCM vs. Healthy)	215 (162 UP, 53 DOWN)	15 (11 UP, 4 DOWN)
Regional Overlap (LV vs. LA)	714 (219 UP, 495 DOWN)	18 (6 UP, 12 DOWN)

DOWN: downregulation; HCM: Hypertrophic cardiomyopathy; LA: Left atrium; LV: Left ventricle; UP: upregulation.

## Data Availability

Data relevant for reported results are provided in Supplementary Material. Sequencing data used for the current study was accessed on 7 May 2025 (https://www.ncbi.nlm.nih.gov/geo/query/acc.cgi?acc=GSE275971). These contain yet unpublished data that are used by the authors for ongoing studies that will be published in the near future and therefore currently not publicly available. Full access to the dataset will be provided at that point.

## Supplementary Materials

The following supporting information can be downloaded at: https://www.mdpi.com/article/10.3390/ijms26146764/s1.

## Abbreviations

The following abbreviations are used in this manuscript:

DEG	Differentially expressed genes
DEM	Differentially expressed microRNAs
HCM	Hypertrophic cardiomyopathy
hLA	Healthy left atrium
hLV	Healthy left ventricle
LA	Left atrium
LV	Left ventricle
mReg	Master regulator
PW	Pathway
upReg	Upstream regulator
